# Supplementary material for: Targeting therapy-resistant lung cancer stem cells via disruption of the AKT/TSPYL5/PTEN positive-feedback loop
Source: Commun Biol. 2021 Jun 23;4:778. doi: 10.1038/s42003-021-02303-x (PMC8222406; doi:10.1038/s42003-021-02303-x)
Supplement: Supplementary file 2 — Supplementary Information [file 42003_2021_2303_MOESM2_ESM.pdf]

Targeting therapy-resistant lung cancer stem cells via disruption of the AKT/TSPYL5/PTEN positive-feedback loop

In-Gyu Kim[1,2]*, Jei-Ha Lee[1], Seo-Yeon Kim[1], Chang-Kyu Heo[3], Rae-Kwon Kim[1], and Eun-Wie Cho[3]*

[1]Department of Radiation Biology, Environmental Radiation Research Group, Korea Atomic Energy Research Institute, 111 Daedeok-daero 989 Beon-gil, Yuseong-gu, Daejeon 34057, Korea
[2]Department of Radiation Biotechnology and Applied Radioisotope, Korea University of Science and Technology, 111 Daedeok-Daero 989 Beon-gil, Yuseong-gu, Daejeon 34057, Korea
[3]Rare Disease Research Center, Korea Research Institute of Bioscience and Biotechnology, 125 Gwahak-ro, Yuseong-gu, Daejeon 34141, Korea

1. Supplementary Figures

Supplementary Figure 1. TSPYL5 as well as CSC-associated factors were increased in fractionated γ-radiation-exposed cells

Supplementary Figure 2. Cancer stemess related properties of ALDH1 (ALDH1A1 or ALDH1A3)-suppressed A549 or ALDH1-overexpressing H460 cells

Supplementary Figure 3. RT-PCR assay of ALDH1, CD44, PTEN and TSPYL5 expression in sorted $ALDH1^{high}$ and $ALDH1^{low}$ A549 cells

Supplementary Figure 4. AKT inhibitor (MG2206, 20μM) suppressed sphere-forming ability of A549 cells

Supplementary Figure 5. The intracellular distributions and the level of ALDH1 and CD44 by TSPYL5 mutations

Supplementary Figure 6. EMT and CSC-associated characteristics of TSPYL5-overexpressing H460 cells

Supplementary Figure 7. *In vivo* SUMOylation assay of H460 cells transfected with His_6-SUMO1 and TSPYL5 or related mutants (T177A, T326A, and T409A)

Supplementary Figure 8. Chromatin immunoprecipitation (ChIP) assay of ALDH1, CD44, and PTEN in different promoter sites using the TSPYL5 antibody

Supplementary Figure 9. TS120-T peptide showed no effects on CSC-like properties of H460 cells, which express endogenous TSPYL5 at a low level

Supplementary Figure 10. Immunohistochemical assay of TSPYL5, CD44, and PTEN expression

Supplementary Figure 11. Therapy-resistance of lung cancer cells depending on TSPYL5 expression

Supplementary Figure 12. EMT potential and cell growth of various tumor cell lines depending on the level of TSPYL5

Supplementary Figure 13. Uncropped Western blot images

2. Supplementary Tables

Supplementary Table 1. Prediction of potential phosphorylation sites in TSPYL5

Supplementary Table 2. Primer for expression vector construction

Supplementary Table 3. Mutagenesis primer sequences

Supplementary Table 4. List of antibodies used in this study

Supplementary Table 5. siRNA sequences

Supplementary Table 6. Primers for RT-PCR

Supplementary Table 7. Primers for ChIP assays

1. Supplementary Figures

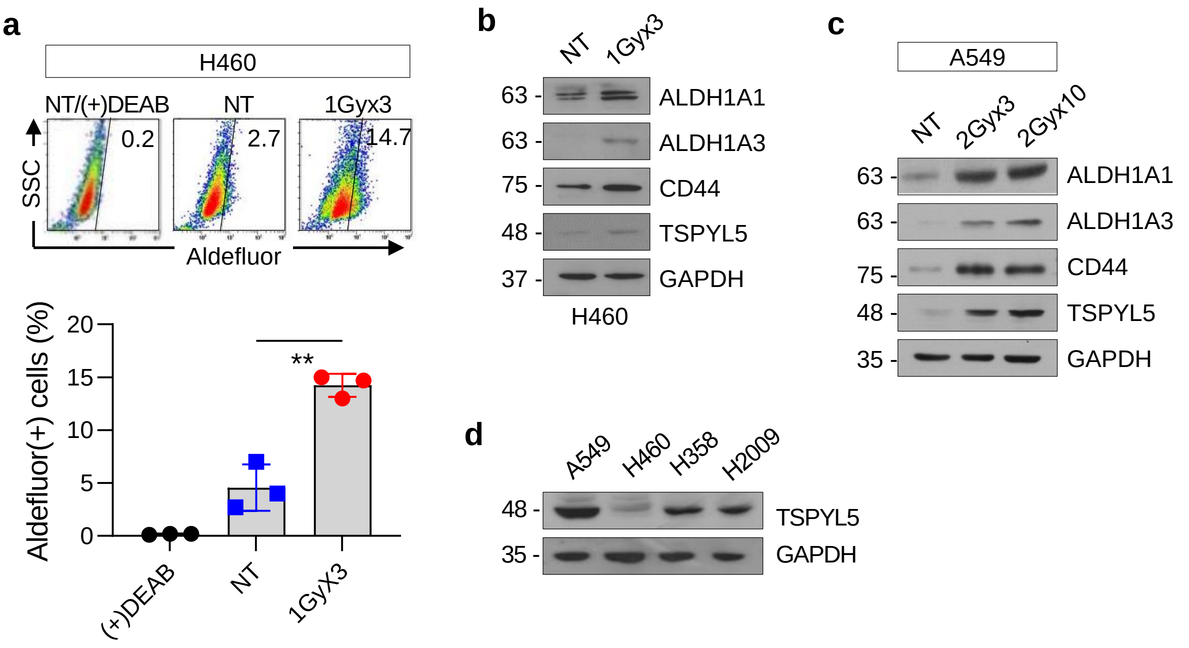

Supplementary Figure 1. TSPYL5 as well as CSC-associated factors were increased in fractionated γ-radiation-exposed cells. a Flow cytometric analysis of ALDH1 activity (ALDEFLUOR assay) in fractionated γ-radiation-exposed (1Gy×3) H460 cells. N,N-diethylaminobenzaldehyde (DEAB)-treated cells were used as negative controls for gating. Numbers in the upper right represent the percent of positive cells. *n* = 3 independent experiments. NT: not treated. **b** Cellular levels of CSC-associated factors (ALDH1, CD44) and TSPYL5 in fractionated γ-radiation-exposed (1Gy x 3) H460 cells shown by Western blot analysis. **c** Western blot analysis of cellular ALDH1, CD44 and TSPYL5 levels in fractionated γ-radiation-exposed (2Gy x 10 with a 3 day interval, total of 20 Gy) A549 cells. **d** Cellular levels of TSPYL5 in various lung cancer cell lines.

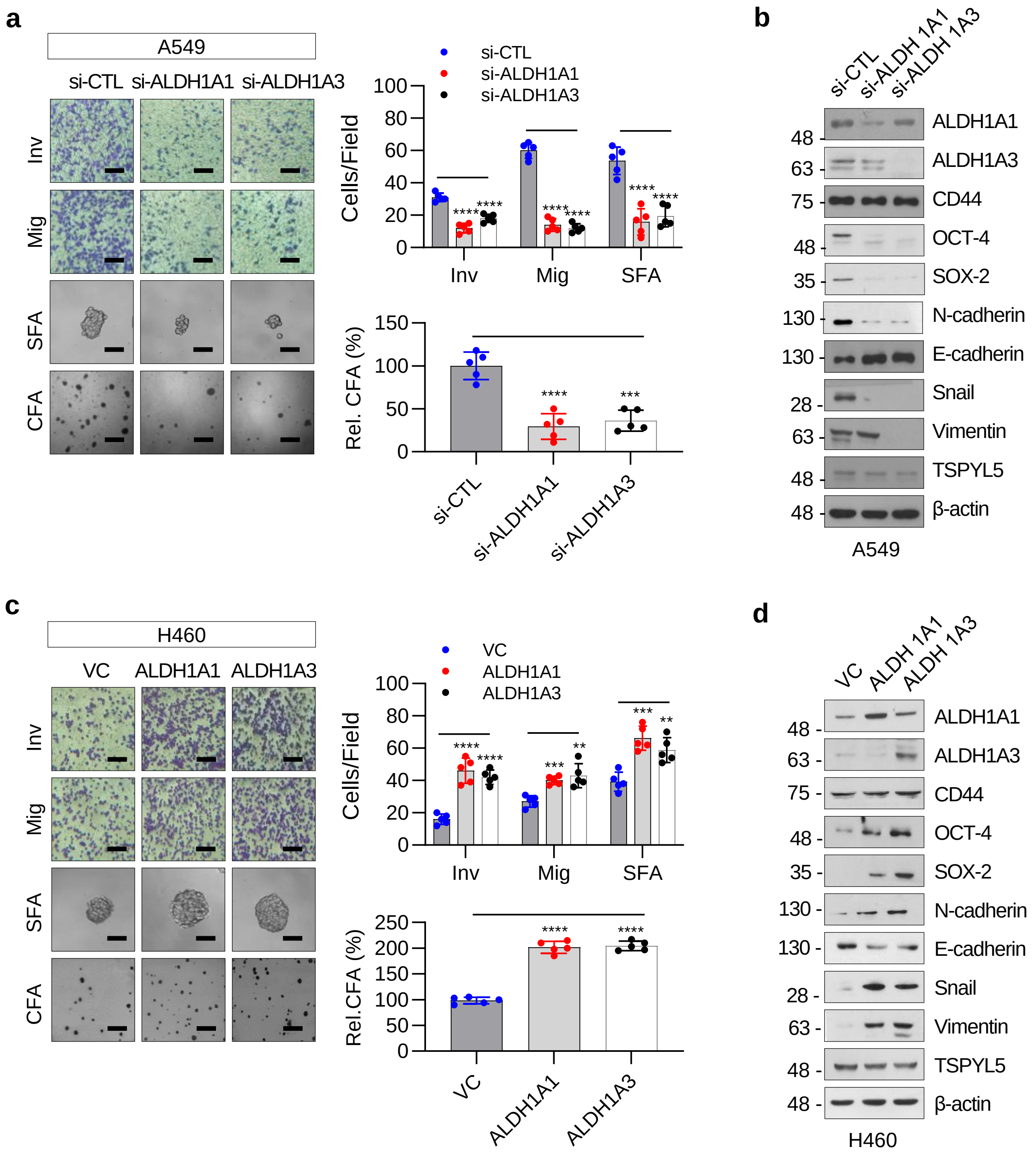

Supplementary Figure 2. Cancer stemess related properties of ALDH1 (ALDH1A1 or ALDH1A3)-suppressed A549 or ALDH1-overexpressing H460 cells. a Invasion/migration, sphere-forming and colony-formation ability of ALDH1 (ALDH1A1 or ALDH1A3)-suppressed A549 cells. *n* = 5. **b** Expression of EMT and CSC biomarkers in ALDH1-suppressed A549 cells shown by Western blot analysis. **c** Invasion/migration, sphere-forming and colony-formation ability of ALDH1-overexpressing H460 cells. *n* = 5. **d** EMT and CSC biomarkers in ALDH1-overexpressing H460 cells shown by Western blot assay. Data represent mean ± s.d. using two-tailed t-test. $^{**}p < 0.01$, $^{***}p < 0.001$, $^{****}p < 0.0001$. Scale bar: 20 μm.

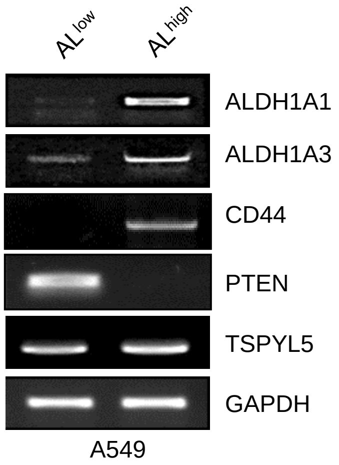

Supplementary Figure 3. RT-PCR assay of ALDH1, CD44, PTEN and TSPYL5 expression in sorted ALDH1^{high} and ALDH1^{low} A549 cells.

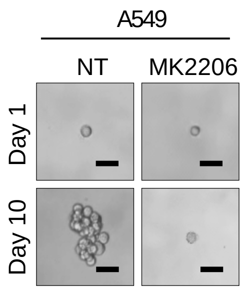

Supplementary Figure 4. AKT inhibitor (MG2206, 20μM) suppressed sphere-forming ability of A549 cells. Scale bar: 20 μm.

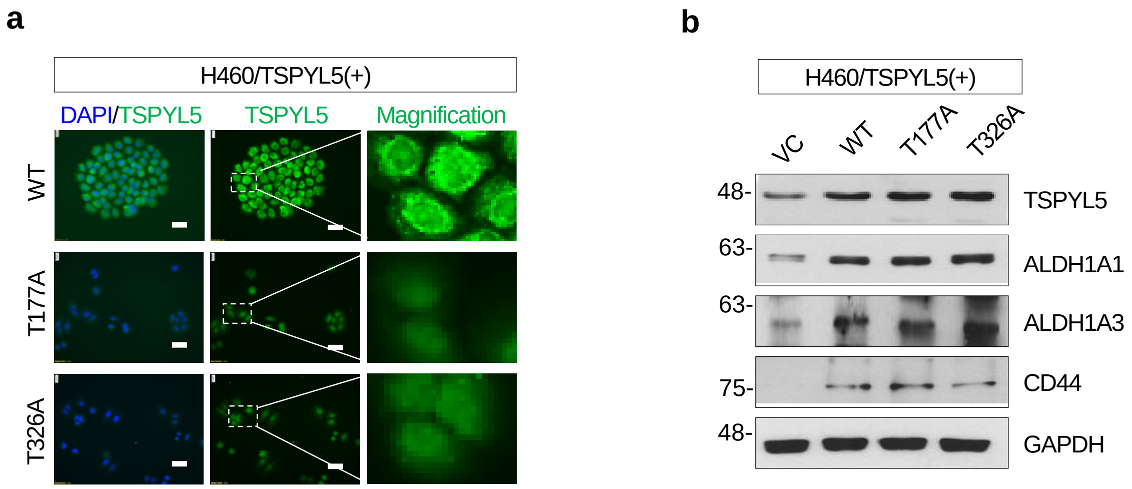

Supplementary Figure 5. The intracellular distributions and the level of ALDH1 and CD44 by TSPYL5 mutations. a Alanine substitutions at threonine-177 or threonine-326 in TSPYL5 showed no influences on nuclear translocation of TSPYL5. Scale bar: 20 μm. **b** Western blot analysis of cellular ALDH1, CD44 and PTEN levels in T177A-TSPYL5 or T326A-TSPYL5 transfected H460 cells.

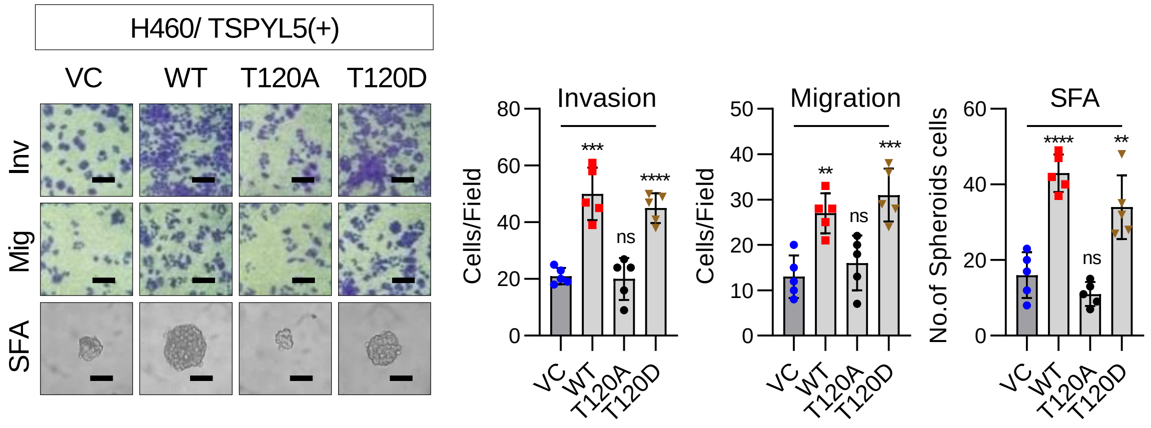

Supplementary Figure 6. EMT and CSC- associated characteristics of TSPYL5-overexpressing H460 cells. Invasion/migration and sphere-formation abilities of TSPYL5-overexpressing H460 cells were abolished by T120A mutation; however, T120D-mutant, a mimic of phosphorylated TSPYL5, maintained the characteristics of WT-TSPYL5. n = 5 independent experiments for each group. Data represent mean ± s.d. using a two-tailed t-test. ns: not statistically significant, *p <0.05, **p < 0.01, ***p < 0.001, ****p <0.0001. Scale bar: 20 μm

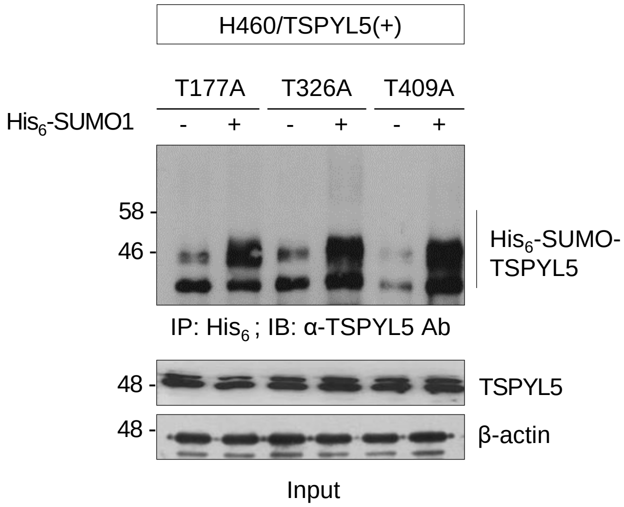

Supplementary Figure 7. ***In vivo*** **SUMOylation assay of H460 cells transfected with His$_6$-SUMO1 and TSPYL5 or related mutants (T177A, T326A, and T409A).** Cell lysates were precipitated with Ni-agarose bead and Western blot analysis was performed with anti-TSPYL5 antibody. Whole cell lysates were also analysed for TSPYL5 and β-actin.

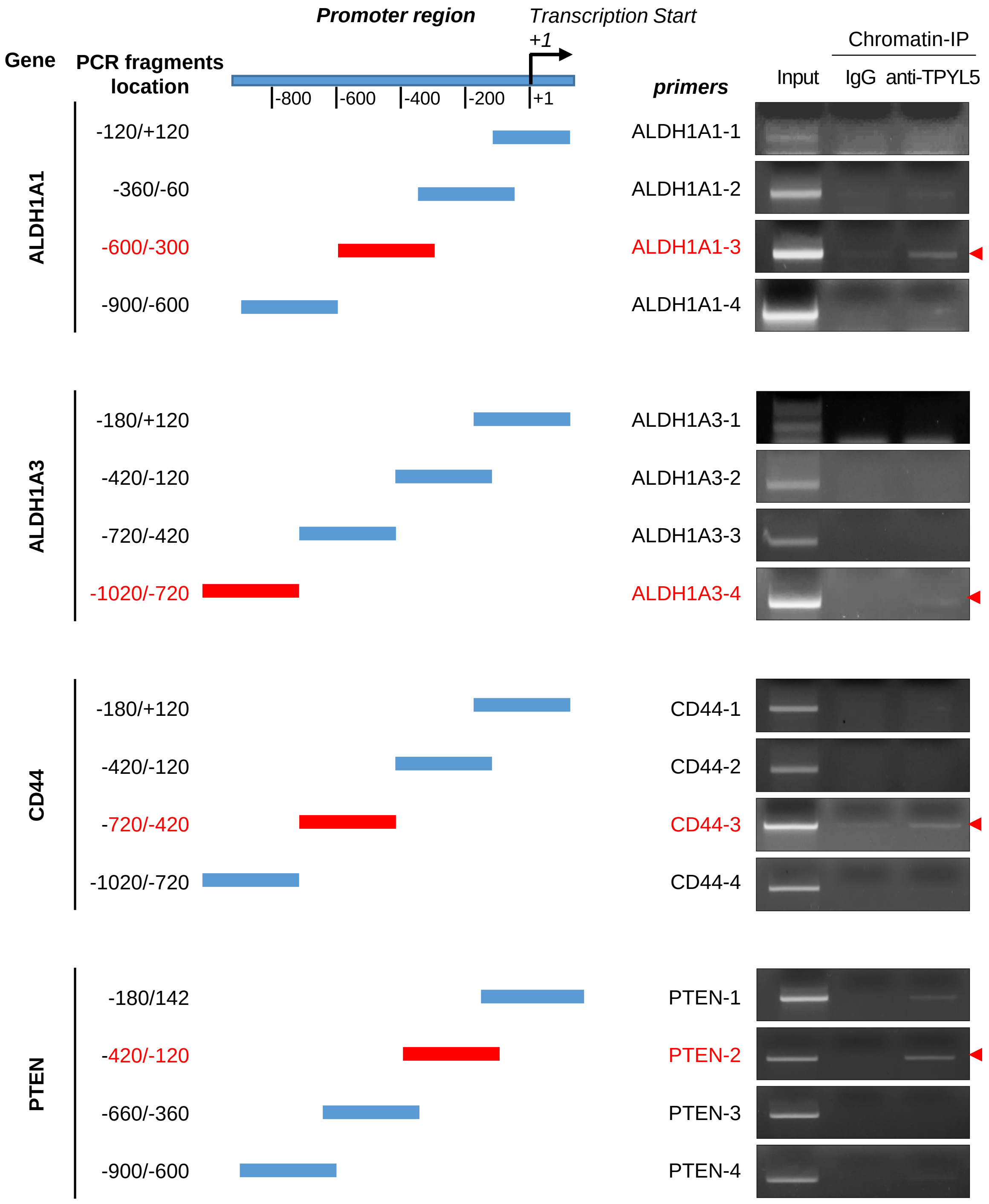

Supplementary Figure 8. Chromatin immunoprecipitation (ChIP) assay of ALDH1, CD44, and PTEN in different promoter sites using the TSPYL5 antibody. Promoter primer sequences are listed in Supplementary Table 6.

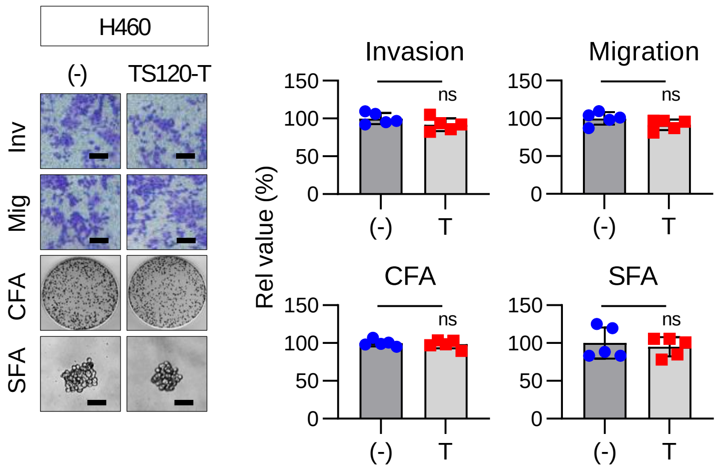

Supplementary Figure 9. TS120-T peptide showed no effects on CSC-like properties of H460 cells, which express endogenous TSPYL5 at a low level. Self-renewal potential and EMT properties H460 cells treated with TS120-T peptide (10uM) were examined by sphere-forming and invasion/migration assays. *n* = 5. Data represent mean ± s.d. using a two-tailed t-test. ns: not statistically significant. Scale bar: 20 µm.

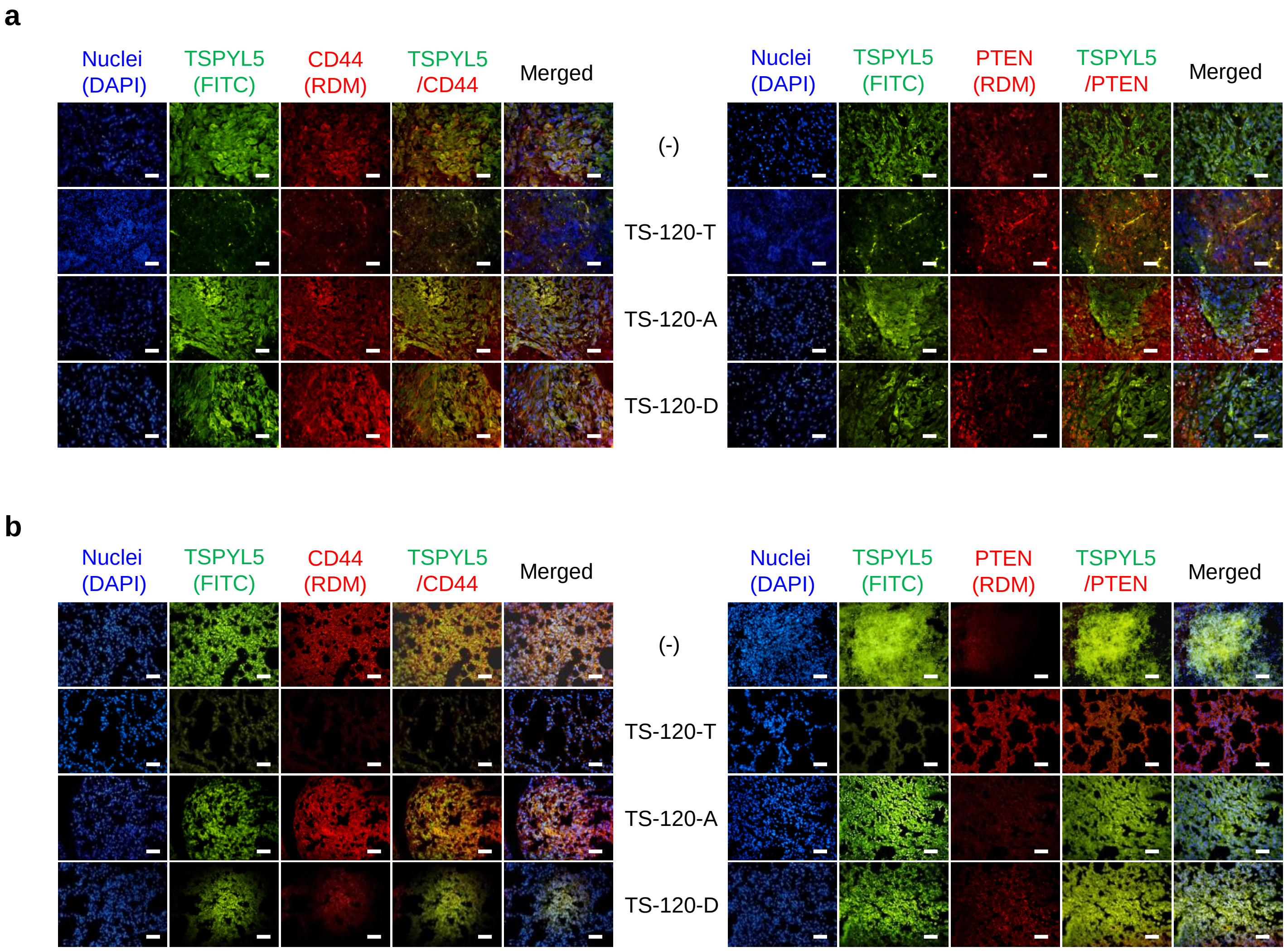

Supplementary Figure 10. Immunohistochemical assay of TSPYL5, CD44, and PTEN expression in (**a**) tumor burden from xenograft model or (**b**) metastatic foci in the lung from metastatic model treated with TS120 peptides (See Figure 8). Original magnifications: ×400. Scale bar: 40 μm.

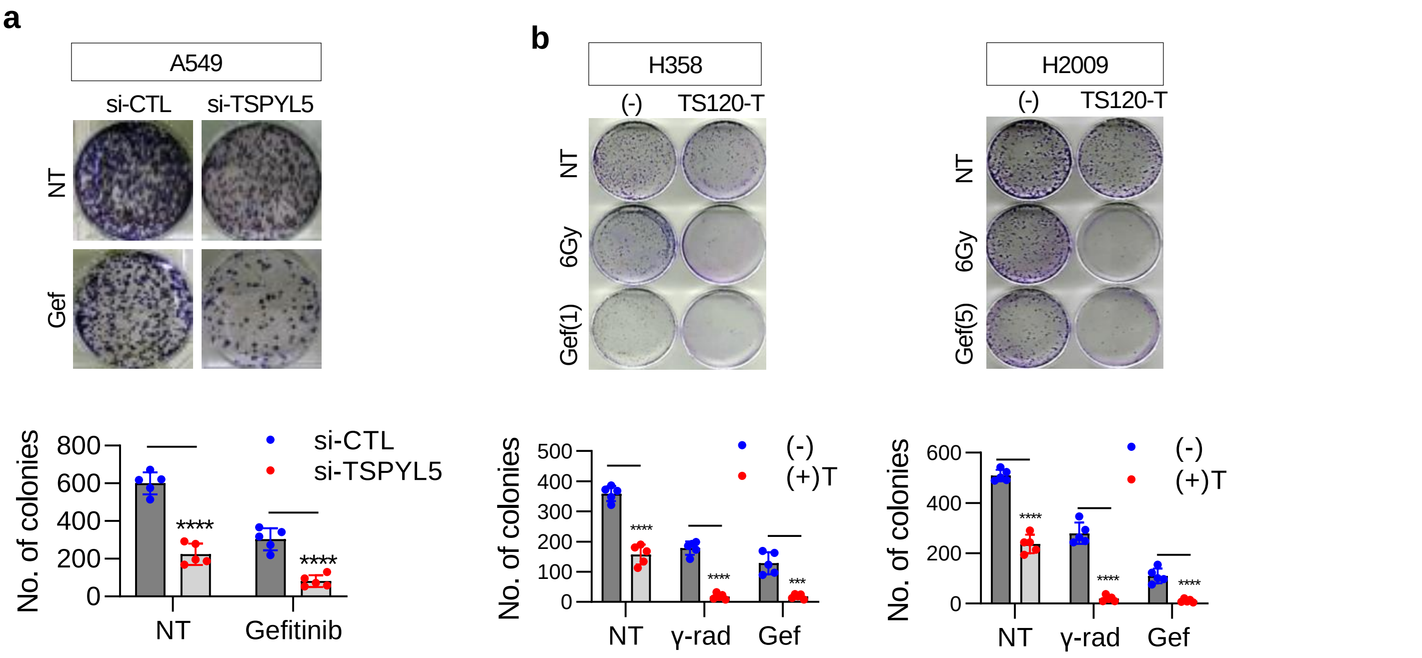

Supplementary Figure 11. Therapy-resistance of lung cancer cells depending on TSPYL5 expression. a Drug-sensitizing effect of TSPYL5-knockdown on A549 cells. TSPYL5-knockdown with si-TSPYL5 sensitized A549 cells to the treatment of gefitinib (1 μM). n = 5. **b** The therapy-sensitizing effect of TS120-T peptide on $\text{TSPYL5}^{\text{high}}$-NSCLC cells. H358 and H2009 cells expressing a high level of TSPYL5 (See in Supplementary Fig.1d) were treated with TS120-T peptide (10 μM) simultaneously with radiation (6 Gy) or gefitinib (1 or 5 μM) and cell survival was examined by colony formation assay. n = 5. Data represent mean ± s.d. using two-tailed t-tests. $^{*}p < 0.05$, $^{**}p < 0.01$, $^{***}p < 0.001$, $^{****}p < 0.0001$.

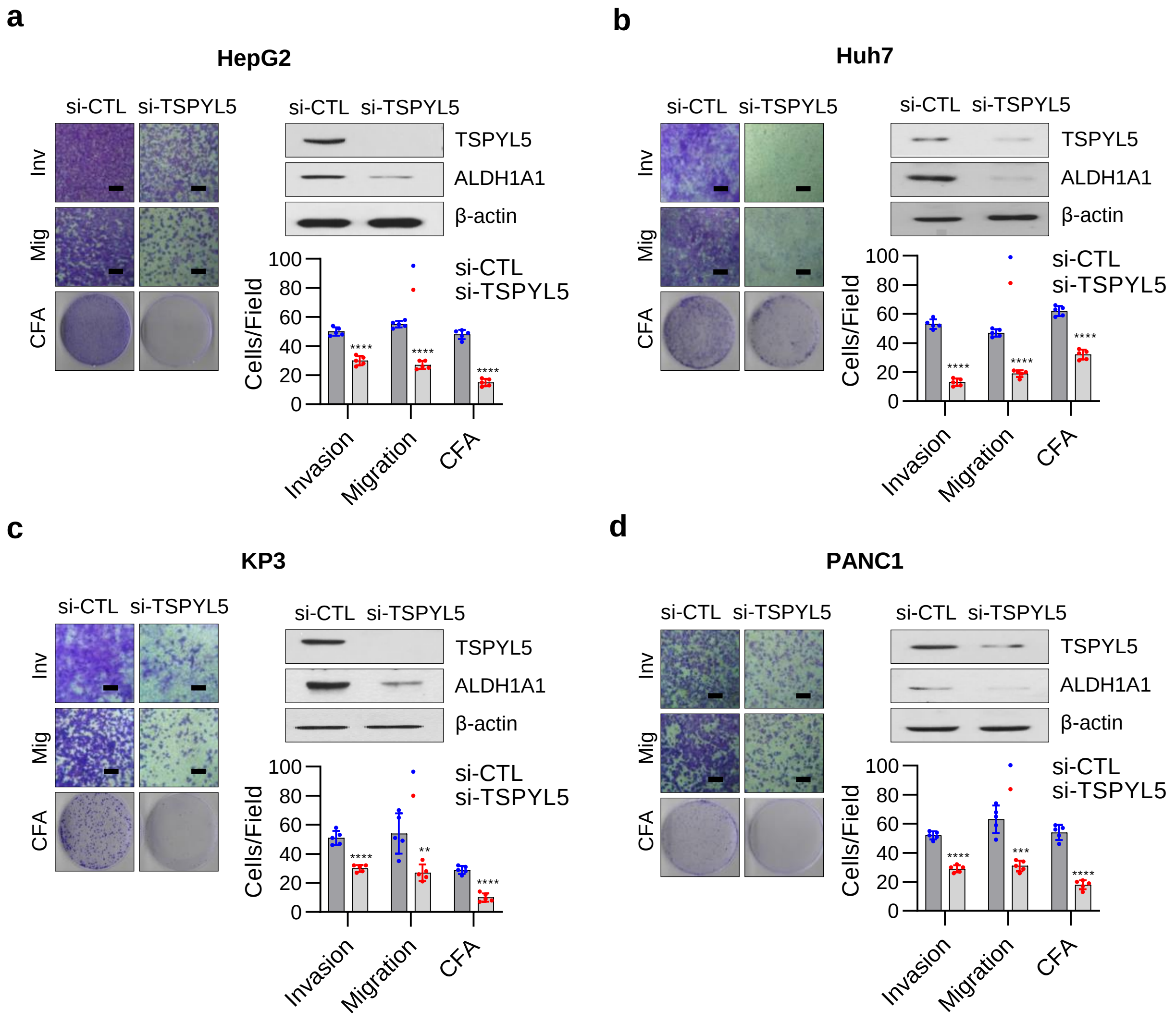

Supplementary Figure 12. EMT potential and cell growth of various tumor cell lines depending on the level of TSPYL5. TSPYL5 in various tumor cell lines were knockdown with TSPYL5-specific siRNA and cellular properties as well as expression of stemness marker ALDH1 was analyzed. Liver cancer cell lines: HepG2(**a**) and Huh7(**b**); pancreatic cancer cell lines: KP3(**c**) and PANC1(**d**). $n = 5$ independent experiments. Data represent mean ± s.d. using two-tailed t-tests. $^{*}p < 0.05$, $^{**}p < 0.01$, $^{***}p < 0.001$, $^{****}p < 0.0001$. Scale bar: 20 μm

Supplementary Figure 13. Uncropped Western blot images

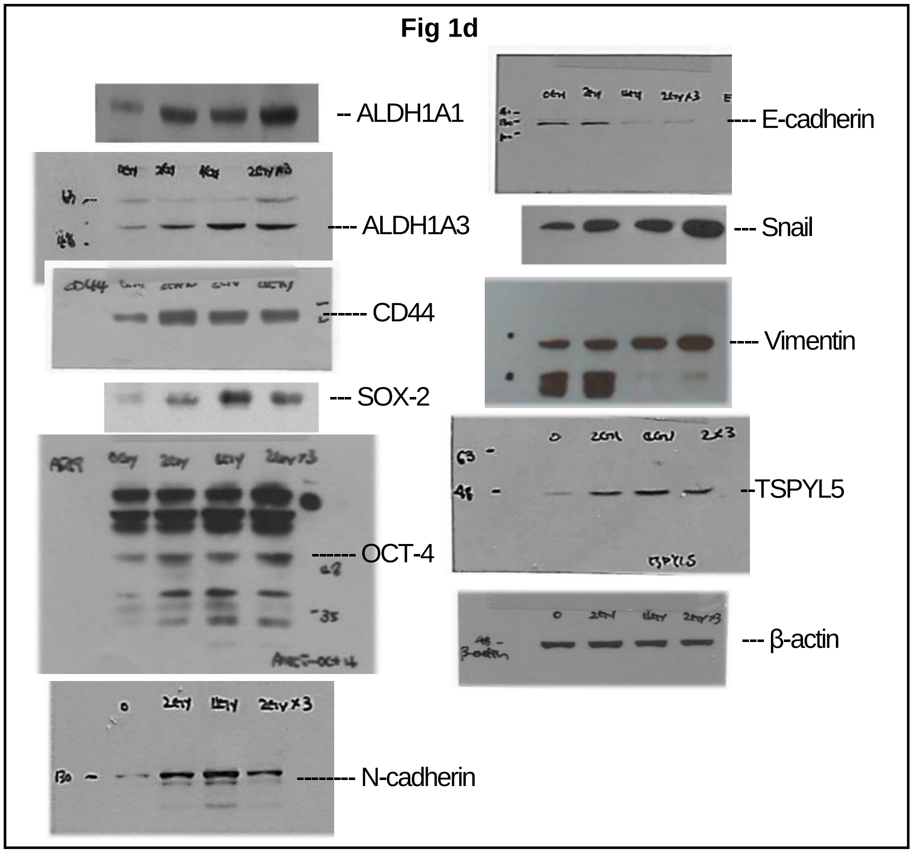

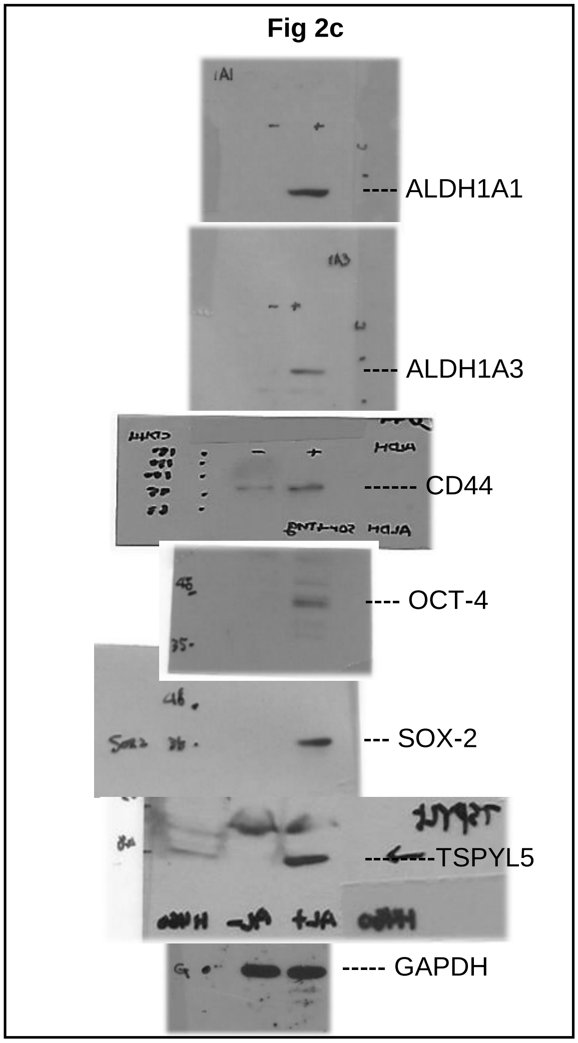

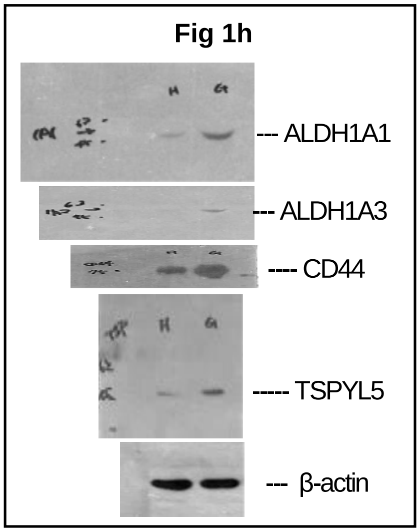

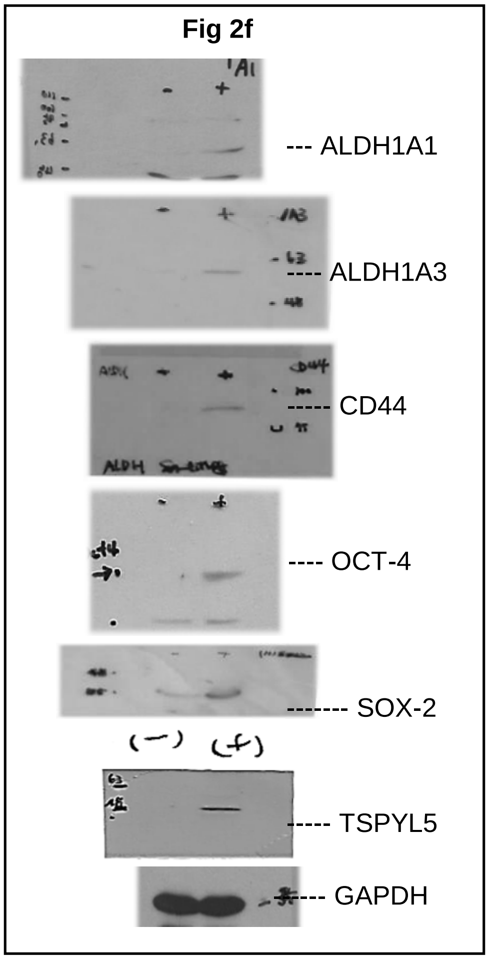

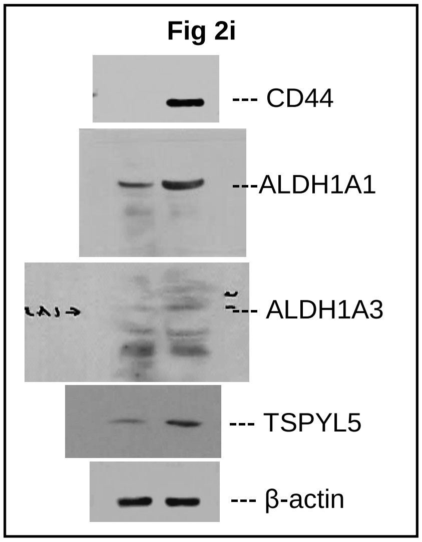

Fig 3b

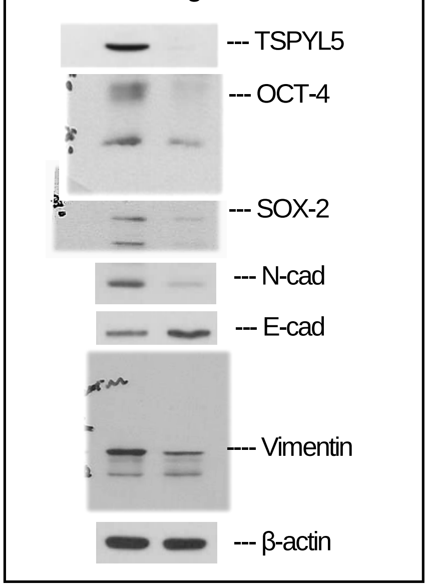

Fig 3d

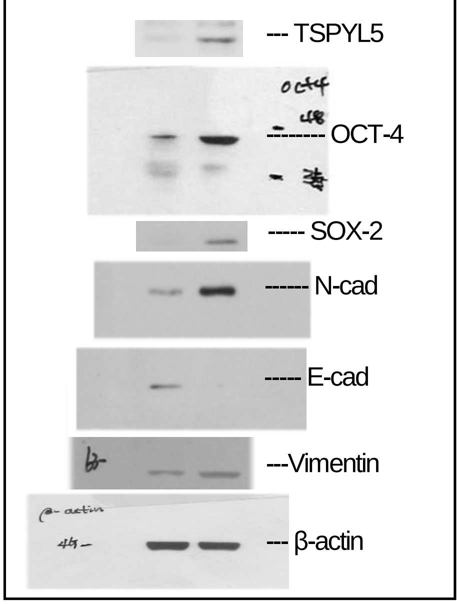

Fig 3e

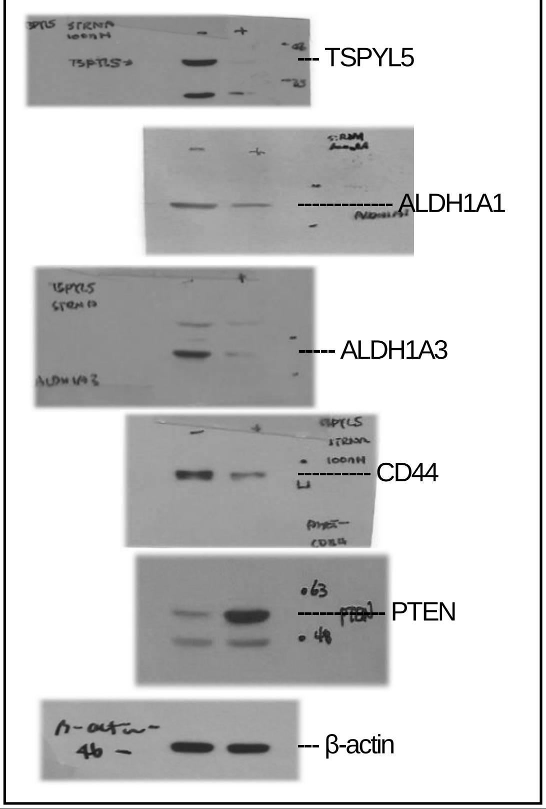

Fig 3f

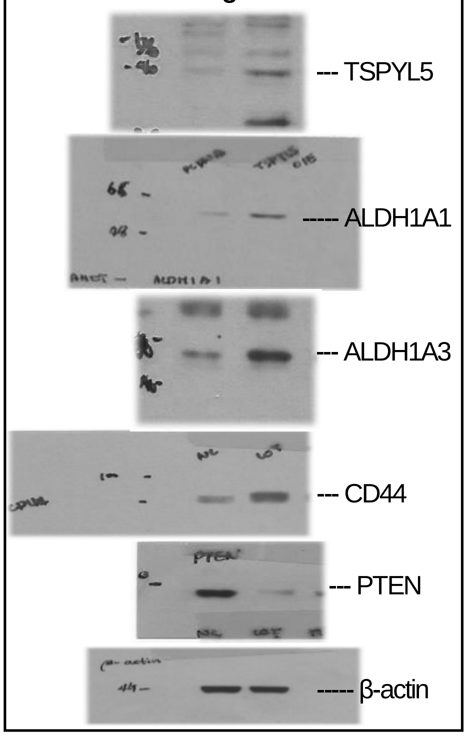

Fig 4a

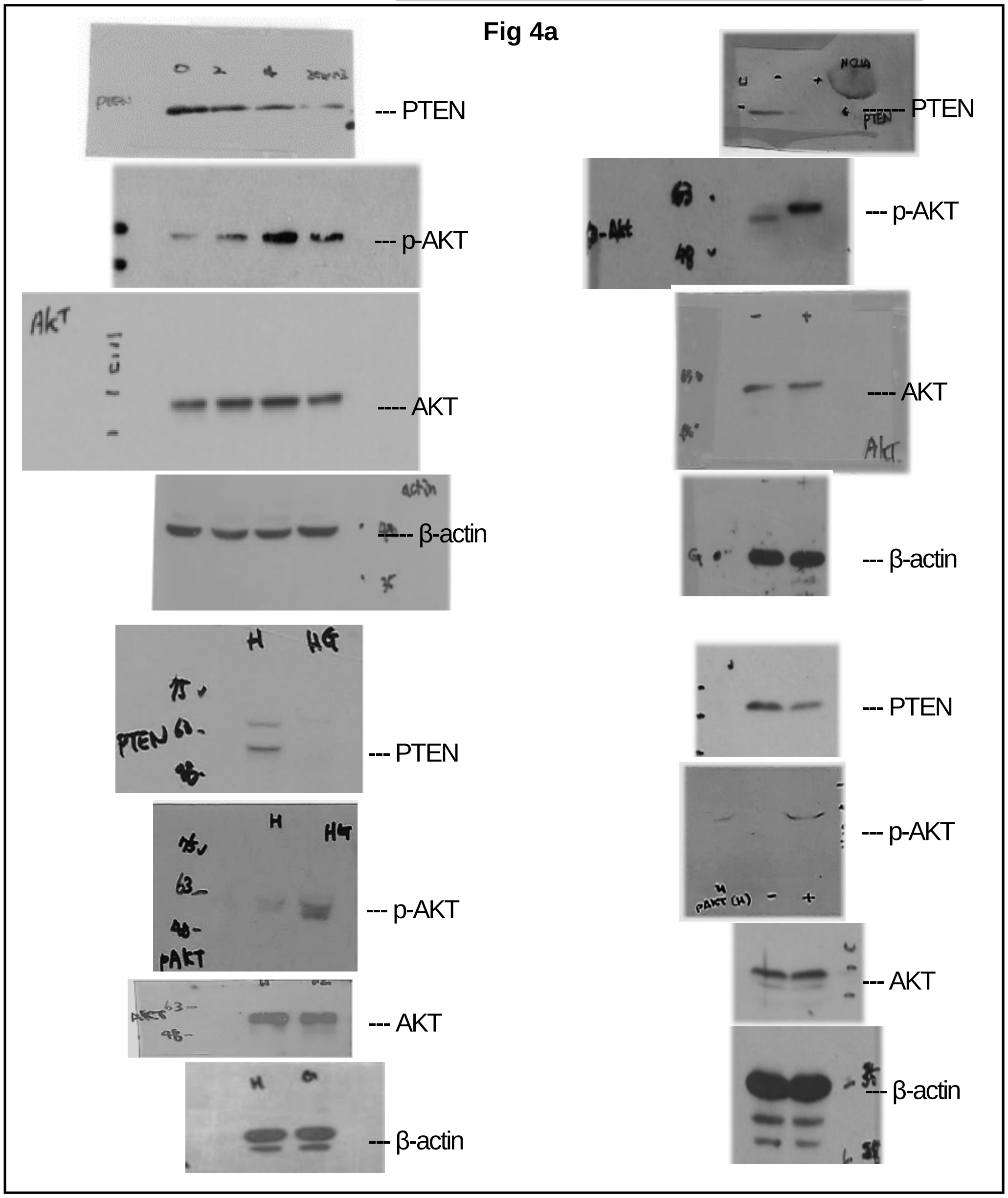

Fig 4b

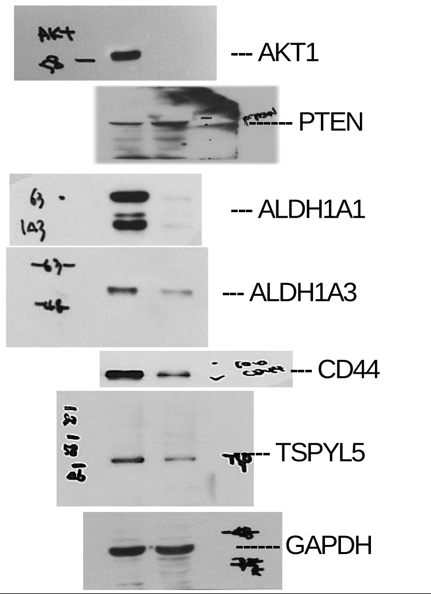

Fig 4c

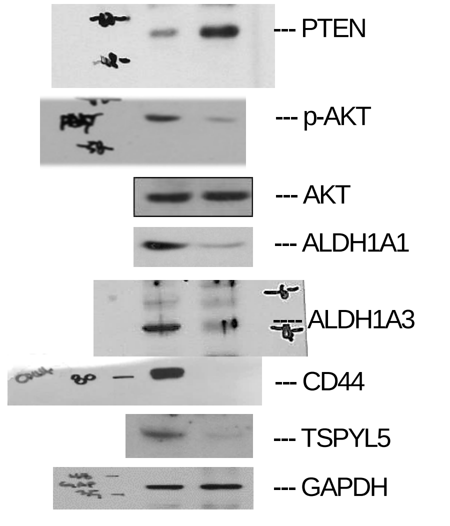

Fig 4d

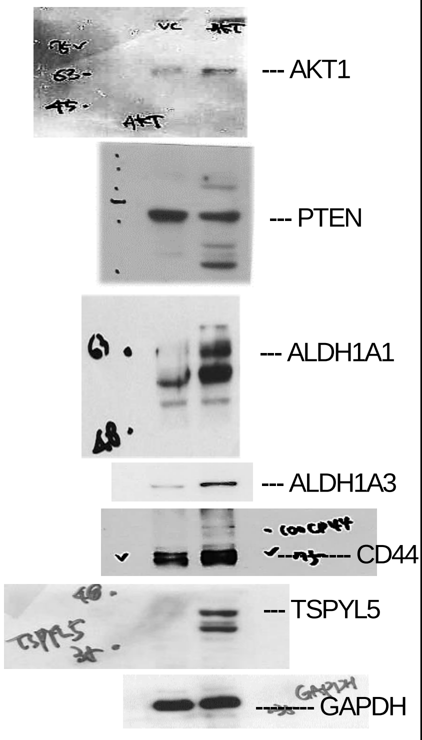

Fig 4e

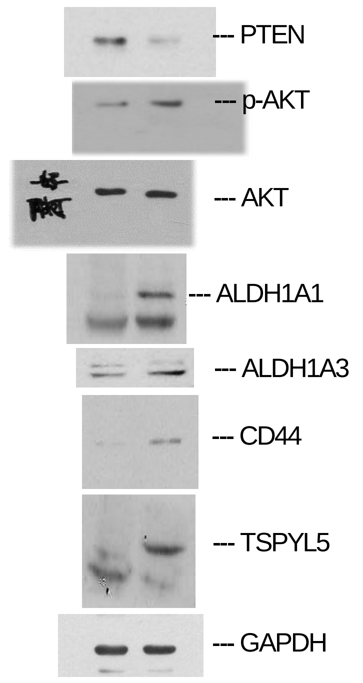

Fig 5b

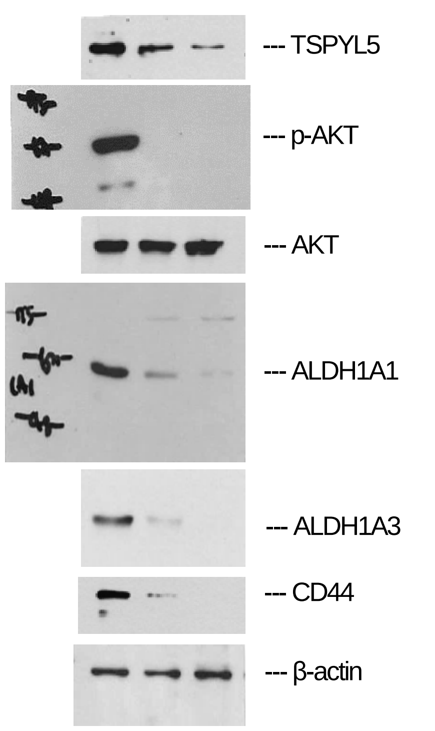

Fig 4f

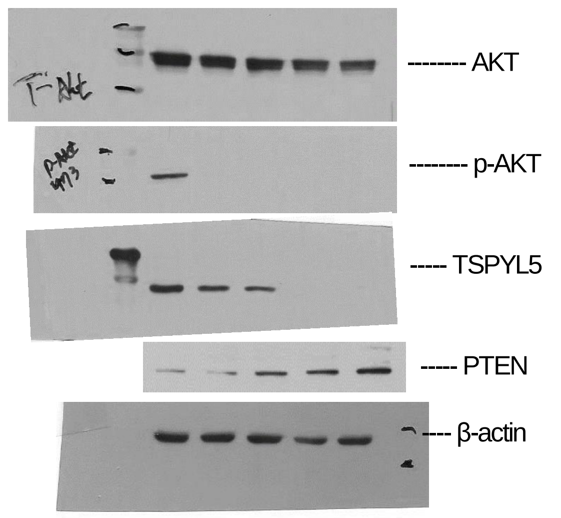

Fig 5e

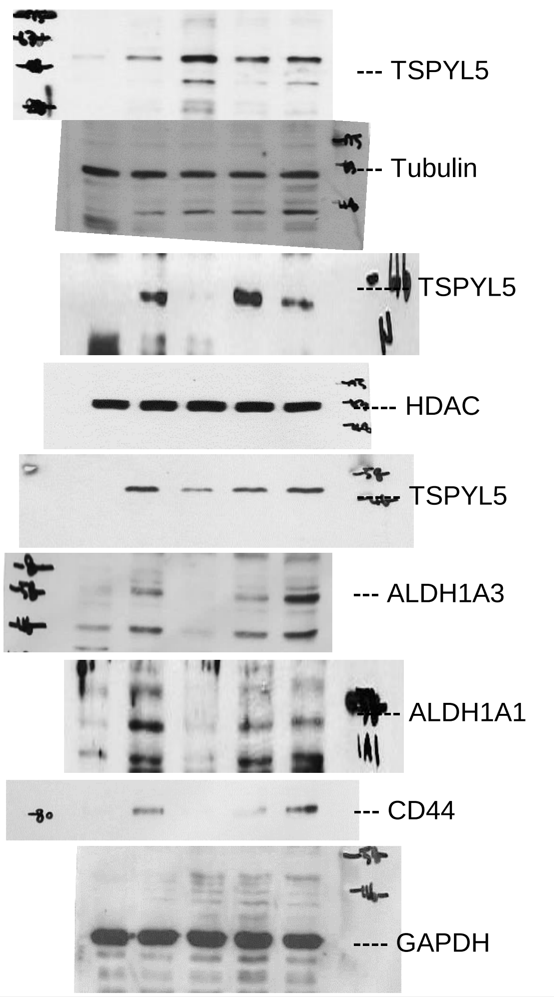

Fig 5f

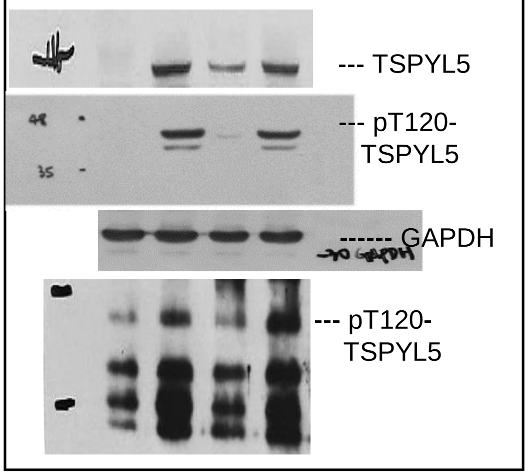

Fig 5g

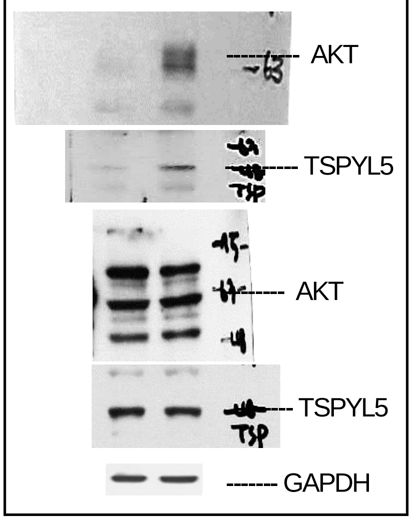

Fig 5h

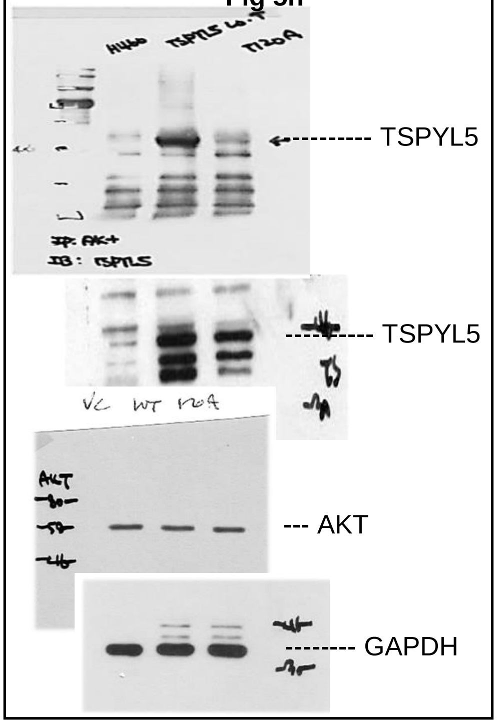

Fig 6a

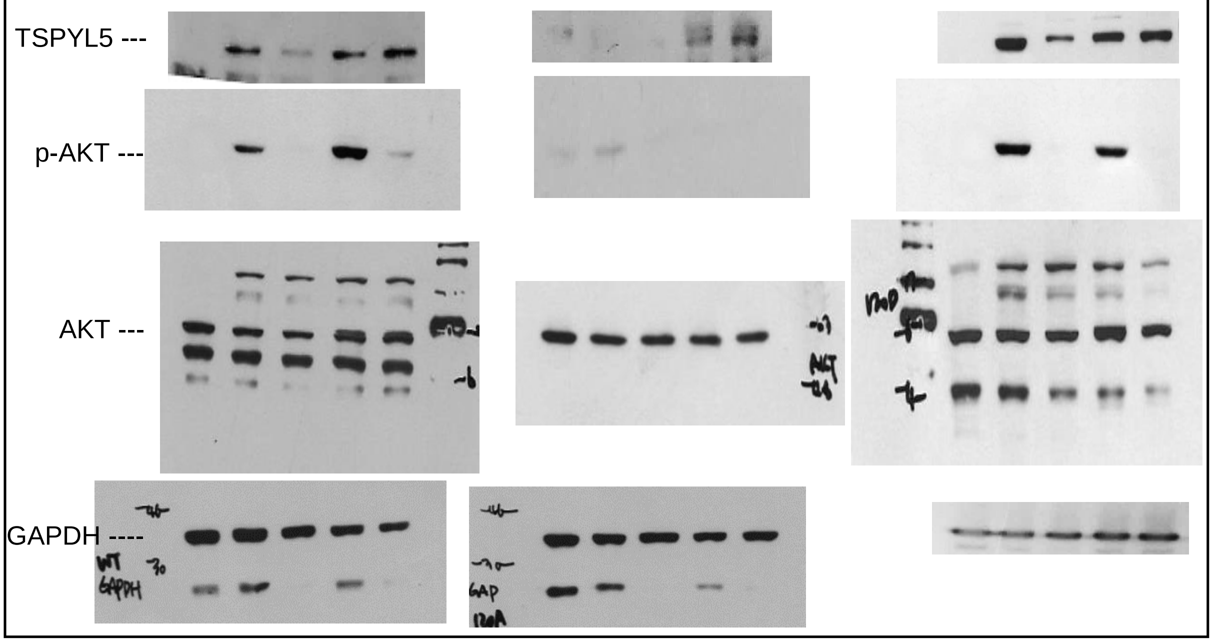

Fig 6b

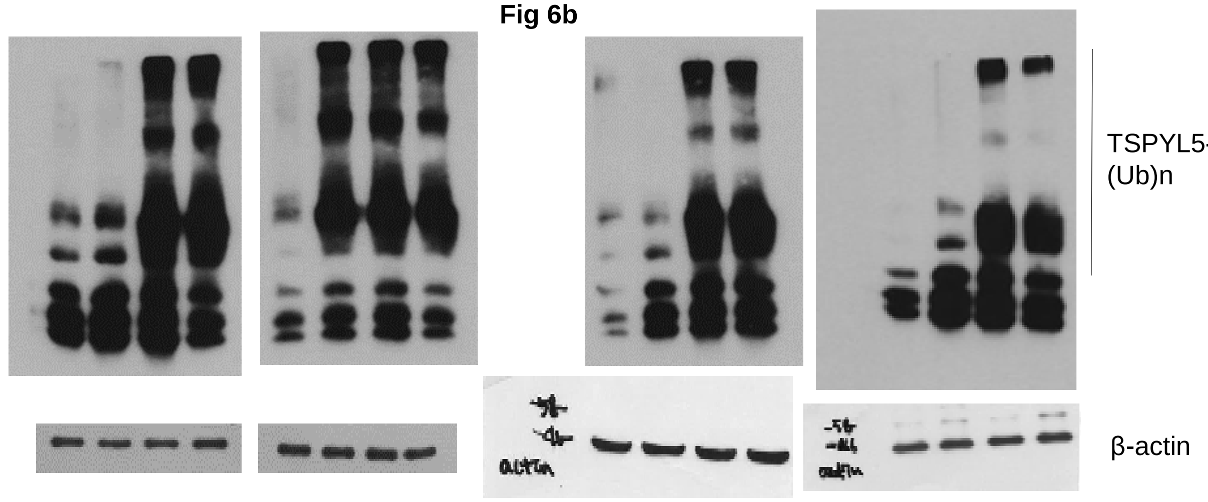

Fig 6d

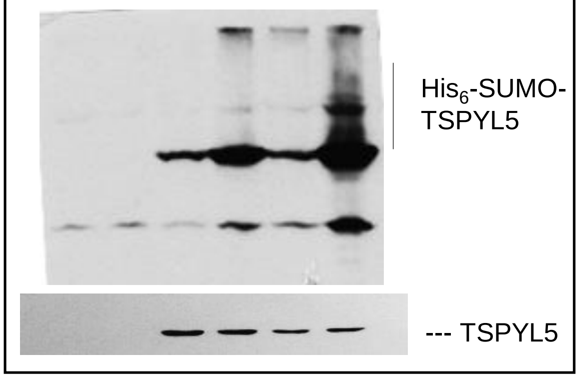

Fig 7c

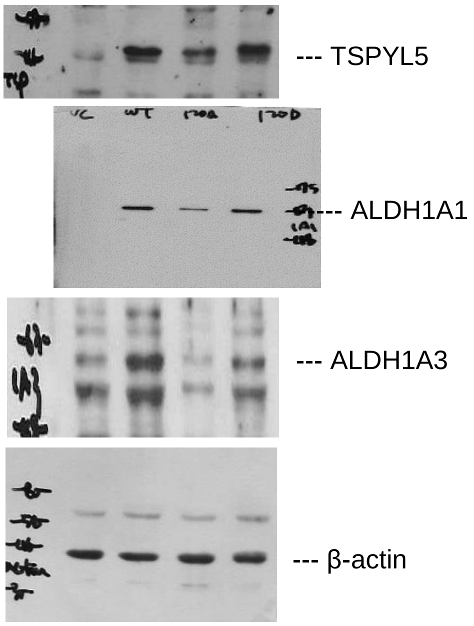

Fig 7d

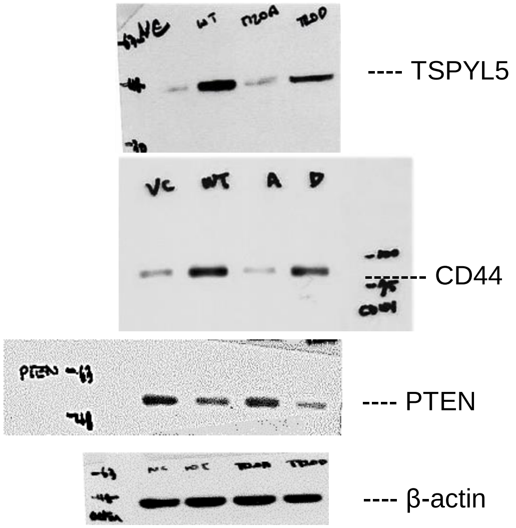

Fig 8d

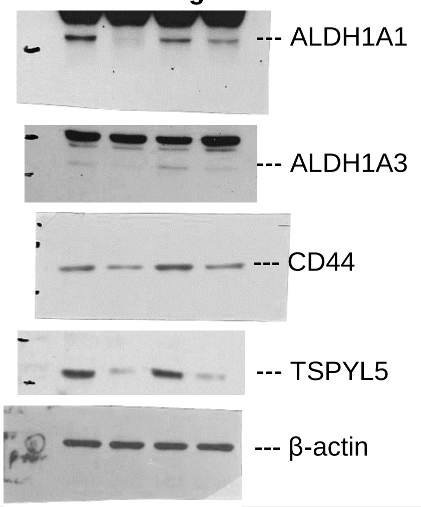

Supple. Fig 1b

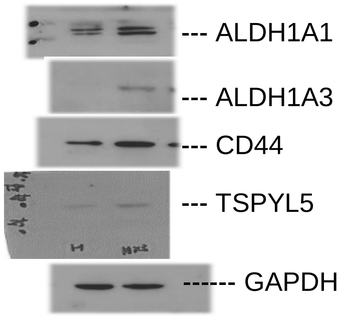

Supple. Fig 1c

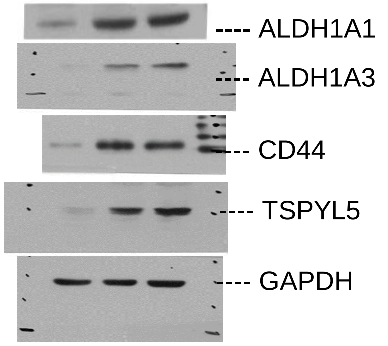

Supple. Fig 2b

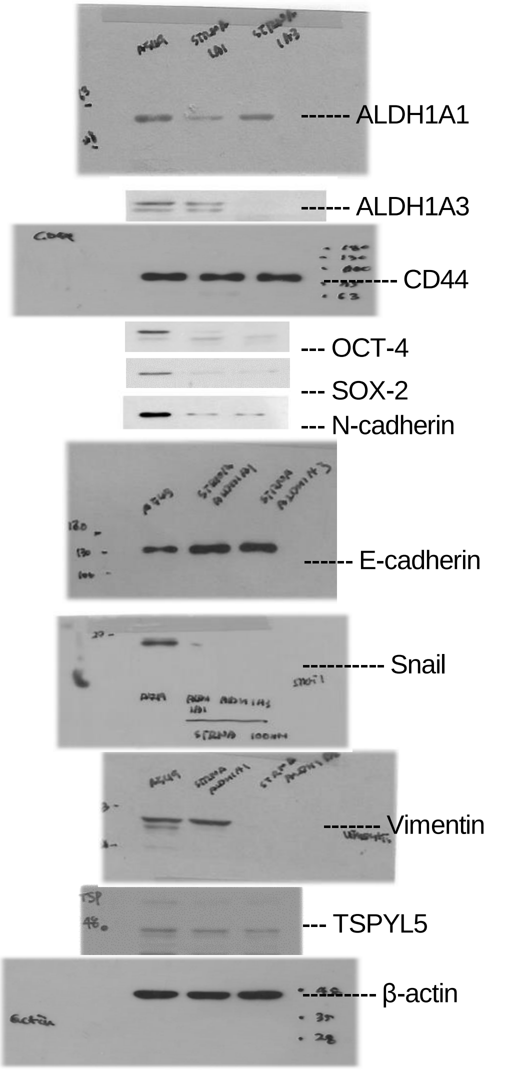

Supple. Fig 1d

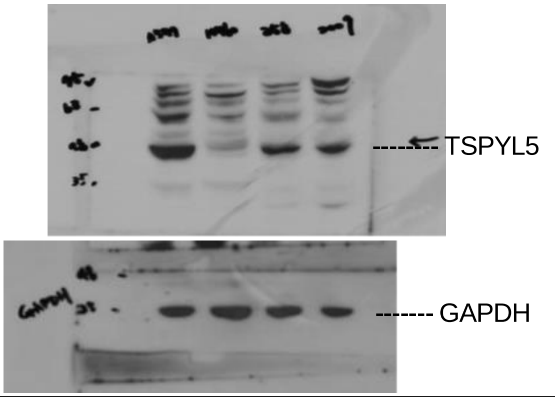

Supple. Fig 2d

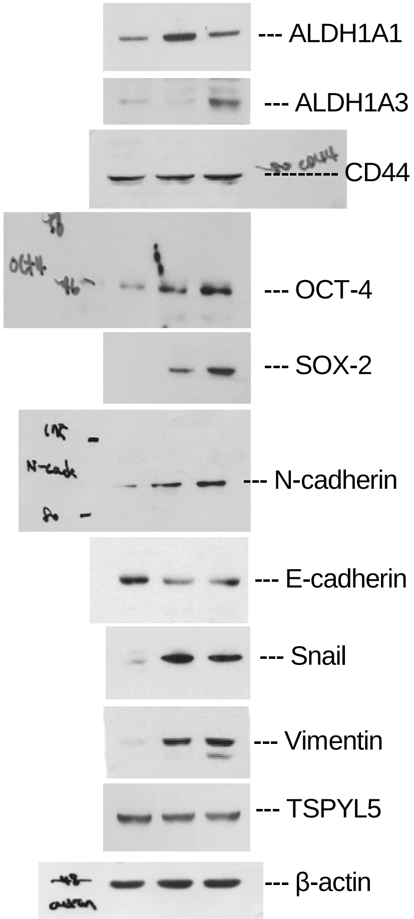

Supple. Fig 5b

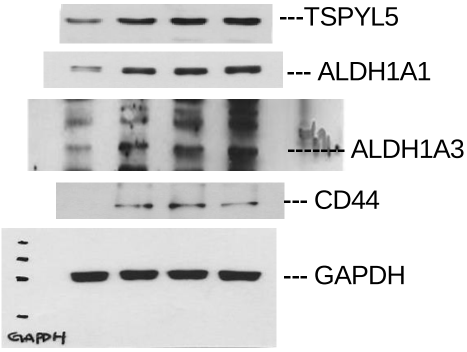

Supple. Fig 7

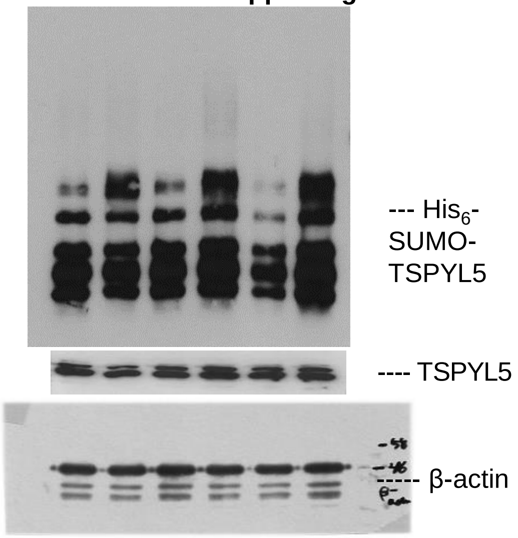

Supple. Fig 12

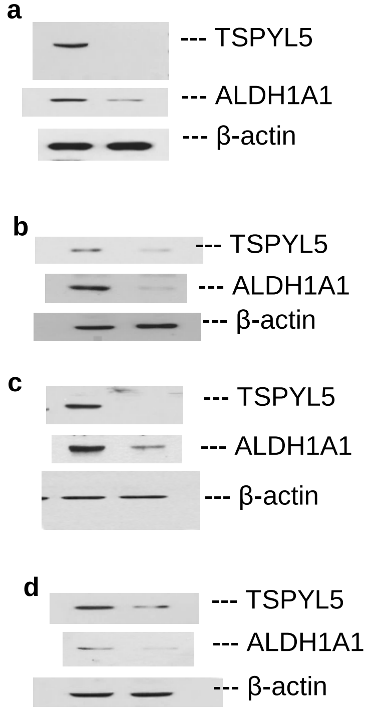

2. Supplementary Tables

Supplementary Table 1. Prediction of potential phosphorylation sites in TSPYL5

Phospho-threonine prediction*			
position	Context	Score	Pred.
48	LGEDTQAAD	0.225	
120	LAADTVFVG	0.566	.T.
125	VFVGTAGTV	0.115	
128	GTAGTVGTV	0.447	
152	KAPETCSTA	0.115	
155	ETCSTAGRG	0.065	
177	AGENTSVSA	0.898	.T.
198	GPPATEGSM	0.088	
204	GSMDTLENW	0.487	
326	VSRSTPIQW	0.803	.T.
408	QPMETTQPG	0.134	
409	PMETTQPGV	0.900	.T.

**Phosphorylation sites were predicted using NetPhos 2.0.*

Supplementary Table 2. Primer for expression vector construction

Target gene		Primer sequences* (5'→ 3')
TSPYL5 (NM_033512.3)	forward	cttaagcttATGAGCGG CCGAAGTCGG
	reverse	tggaattcGTGTTGGATTGGCTCACCCC
ALDH1A1 (NM_000689.5)	forward	atataagcttATGTCATCCTCAGGCACGCC
	reverse	atatgaattcTTATGAGTTCTTCTGAAGATTTTC
ALDH1A3 (NM_000693.4)	forward	atataagcttATGGCCA CCGCTAACGGGGC
	reverse	atatgaattcTCAGGGGTTCTTGTCGCCAAG
AKT1 (NM_005163.2)	forward	atataagcttATGAGCGACGTGGCTATTG
	reverse	tggaattcTCAGGCCGTGCCGCTG GCCG
PTEN (NM_000314.8)	forward	cttaagcttATGACAGCCATCATCAAAG
	reverse	tggaattcGTGACTTTTGTAATTTGTGTA

*Primer sequences containing *Hind* III and *EcoR* I sites, which were written in lower case.

Supplementary Table 3. Mutagenesis primer sequences*

Mutation site	Mutagenesis primer sequences (5'→ 3')**	
**T120A**	forward	gagcgcctggccgcagacgctgtcttcgtgggaacagc
	reverse	gctgttcccacgaagacagcgtctgcggccaggcgctc
**T120D**	forward	gagcgcctggccgcagacgatgtcttcgtgggaacagc
	reverse	gctgttcccacgaagacatcgtctgcggccaggcgctc
**T177A**	forward	ggcggcaggggagaatgcctcggtgtcagctgg
	reverse	ccagctgacaccgaggcattctcccctgccgcc
**T326A**	forward	ggtggtgtctcgttctgctccaatccagtggctc
	reverse	gagccactggattggagcagaacgagacaccacc
**T409A**	forward	gcagccaatggagactgctcagcctggggtgag
	reverse	tcaccccaggctgagcagtctccattggctgc

*Mutation sites were indicated as red characteristic.
**Reference sequence: *TSPYL5* (NM_033512.3)

Supplementary Table 4. List of antibodies used in this study

Full Product Name	Clone Number	Dilution factor	Manufacturer	Catalog Number
TSPYL5 (N-15)	N-15 (affinity purified rabbit pAb)	1:200 (WB) 1:50 (IP)	Santa Cruz Biotechnology.	sc-98186
PTEN (A2B1)	A2B1 (mouse mAb)	1:200 (WB)	Santa Cruz Biotechnology	sc-7974
β-catenin (E-5)	E-5 (mouse mAb)	1:200 (WB)	Santa Cruz Biotechnology	sc-7963
SLUG (A-7)	A-7 (mouse mAb)	1:100 (WB)	Santa Cruz Biotechnology	sc-166476
twist (H-81)	H-81 (rabbit pAb)	1:200 (WB)	Santa Cruz Biotechnology	sc-15393
ZEB1 (H-102)	H-102 (rabbit pAb)	1:100 (WB)	Santa Cruz Biotechnology	sc-25388
CD44 (8E2)	8E2 (mouse mAb)	1:1000 (WB)	Cell Signaling Technology	#5640
Sox2 (D6D9) XP®	D6D9 (rabbit mAb)	1:1000 (WB)	Cell Signaling Technology	#3579
Nanog (1E6C4)	1E6C4 (mouse mAb)	1:1000 (WB)	Cell Signaling Technology	#4893
E-Cadherin (24E10)	24E10 (rabbit mAb)	1:1000 (WB)	Cell Signaling Technology	#3195
SNAIL	(rabbit pAb)	1:1000 (WB)	Abcam	ab63371
Anti-ALDH1A1 antibody	EP1933Y (rabbit mAb)	1:1000 (WB)	Abcam	ab52492
Anti-ALDH1A3 antibody	(rabbit pAb)	1:1000 (WB)	Abcam	ab80176
Anti-Notch2 antibody	(rabbit pAb)	1:500 (WB)	Abcam	ab8926
CD133	(rabbit pAb)	1:200 (WB)	Biorbyt	18124
Anti-OCT-4 [POU5F1]	clone 7F9.2	1:200 (WB)	Millipore.	MAB4305
Anti-N-Cadherin	clone 32/N-Cadherin	1:1000 (WB)	BD Bioscience	610921
Vimentin Antibody	SP20 (rabbit mAb)	1:1000 (WB)	Thermo Fisher Scientific	MA5-16409
CD44-APC	IM7 (rat mAb)	0.06 µg/test	eBioscience™	#17-0441-82
AKT	(rabbit pAb)	1:1000 (WB) 1:50 (IP)	Cell Signaling Technology	#9272
Phospho-AKT (Ser473)	(rabbit pAb)	1:1000 (WB)	Cell Signaling Technology	#9271
Anti-Phospho - (Ser/Thr) Ab	(rabbit pAb)	1:50 (IP)	Abcam	ab117253
Anti-α-Tubulin antibody	clone B-5-1-2 (ascites fluid)	1:4000 (WB)	Sigma-Aldrich	T5168
HDAC1 (10E2)	10E2 (mouse mAb)	1:1000 (WB)	Cell Signaling Technology	#5356
β-actin	C-2 (mouse mAb)	1:200 (WB)	Santa Cruz Biotechnology	sc-8432
GAPDH Antibody (0411)	0411 (mouse mAb)	1:200 (WB)	Santa Cruz Biotechnology	sc-47724

Supplementary Table 5. siRNA sequences

Target gene		siRNA sequences (5'→ 3')
TSPYL5	forward	AAAGGUAGAACUGCAAGGGAUUGGG
	reverse	CCCAAUCCCUUGCAGUUCUACCUUU
ALDH1A1	forward	GAGAGIACGGIIICCAIGA (dTdT)
	reverse	UCAUGGAAACCGUACUCUC (dTdT)
ALDH1A3	forward	CACAGAUGACAACGUCGUA (dTdT)
	reverse	UACGACGUUGUCAUCUGUG (dTdT)
PTEN	forward	GAUAUCAAGAGGAUGGAUU (dTdT)
	reverse	AAUCCAUCCUCUUGAUAUC (dTdT)
AKT1	forward	GACUGACACCAGGUAUUUU (dTdT)
	reverse	AAAUACCUGGUGUCAGUC (dTdT)

Supplementary Table 6. Primers for RT-PCR

Target gene	Primer sequences (5'→ 3')	
TSPYL5	forward	TTCGGCTCTCCAGGAAGTTT
	reverse	GGGGATG GTTCTGAAATGCT
PTEN	forward	TGTGGTCTGCCAGCTAAAGG
	reverse	CACACAGGTAACGGCTGAGG
ALDH1A1	forward	ATAGCCGCATCCAGGATT TT
	reverse	AGAACACTGTGGGCTGGACA
ALDH1A3	forward	GGACTTG GCAGTGGAGTGTG
	reverse	GATCAATCTGAGGCCCCTGT
CD44	forward	CCAATGCCTTTGATGGACCA
	reverse	TGTGAGTGTCCATCTGATTC
AKT	forward	GCACCTTCCATGTGGAGACT
	reverse	CCCAGCAGCTTCAGGTACTC
β-actin	forward	TCAGAAGGATTCCTATGTGGGC
	reverse	CCATCACGATG CCAGTGGTA

Supplementary Table 7. Primers for ChIP assays

Primer	PCR product localization*	Primer sequences (5'→ 3')	
**ALDH1A1-1**	−120/+120	forward	TTACAAATAAGTAGTGTCGTTTT
		reverse	CTTAGTATATTGAATCTTCAAATC
**ALDH1A1-2**	−360/−60	forward	TGATTCCAAGTCTGTCAGAGAAC
		reverse	GGATACGATTGGATGAACAAACTC
**ALDH1A1-3**	−600/−300	forward	ATTTAGGGCTTCTGAGATCACAG
		reverse	ACTTCTCATGCTTTTTAATGCTAC
**ALDH1A1-4**	-900/-600	forward	CAGCTAAATATTAATTTAAGAAC
		reverse	AGTCTTGTGTATTTTCAGTGCTG
**ALDH1A3-1**	−180/+120	forward	CTCCCTTCCGGTCCC GCAGCC
		reverse	CGCGCTCCCTGGCCCGAGGCGCCC
**ALDH1A3-2**	−420/−120	forward	AGGTCTCATGTGCTTTTTTTTAAT
		reverse	GACGCCCGCTGCGCCCCACCCTGC
**ALDH1A3-3**	−720/−420	forward	CCTATCTGAGGATTAAAGCACAGC
		reverse	TAAATGCATAAATTATCACTCGAT
**ALDH1A3-4**	−1020/−720	forward	GCCTCAGCTGTGCACTCCAGGCCA
		reverse	TGGAACAAAGACCGGAGGCACGGA
**CD44-1**	−180/+120	forward	GCTACTTCTTAAAACCTCTGCGGG
		reverse	AAGACCTCGCCCTCTCTCCAGCTC
**CD44-2**	−420/−120	forward	GAGGCTGTAAATAATCGGGGCTGC
		reverse	TCTGGGTTTCACAGGATGTTGGAT
**CD44-3**	−720/−420	forward	GATAGGGCTGGCATTTGGCTCAGC
		reverse	AATGATGGATGAGAAGTTGTATGG
**CD44-4**	−1020/−720	forward	GTAGGAAAGATGGGAGAAAATGAA
		reverse	TAATTTATTCAACCACCTATTCTT
**PTEN-1**	−180/+142	forward	CAAATCTCTGCGAACGATTGTGAT
		reverse	GGATAACGAGCTAAGCCTCGGCCT
**PTEN-2**	−420/−120	forward	TTTGGGCCCTTGAAATTCAACGGC
		reverse	GACTGCATTCGCTCTTTCCTTTTG
**PTEN-3**	−660/−360	forward	CCGGGGCGCGCGGAGCCTGGCCCC
		reverse	AAGCTCTCAGCCGAGCGTGCTGAA
**PTEN-4**	−900/−600	forward	GCAAAGGAAGAAGACGACTTGCCT
		reverse	TTGGCCGCCGTGAAAACCCGGCAG

**Start/end site from transcription start (+1). PCR product sizes are about 300bp.*
