# Supplementary material for: Targeting therapy-resistant lung cancer stem cells via disruption of the AKT/TSPYL5/PTEN positive-feedback loop
Source: Commun Biol. 2021 Jun 23;4:778. doi: 10.1038/s42003-021-02303-x (PMC8222406; doi:10.1038/s42003-021-02303-x)
Supplement: Supplementary file 3 — Description of Additional Supplementary Files [file 42003_2021_2303_MOESM3_ESM.pdf]

Description of Additional Supplementary Files

File Name: Supplementary Data 1
Description: Source data for graphs
