# Supplementary material for: Targeting therapy-resistant lung cancer stem cells via disruption of the AKT/TSPYL5/PTEN positive-feedback loop
Source: Commun Biol. 2021 Jun 23;4:778. doi: 10.1038/s42003-021-02303-x (PMC8222406; doi:10.1038/s42003-021-02303-x)
Supplement: Supplementary file 5 — Reporting Summary [file 42003_2021_2303_MOESM5_ESM.pdf]

nature research

Corresponding author(s): Eun-Wie Cho and In-Gyu Kim

Last updated by author(s): May 22, 2021

Reporting Summary

Nature Research wishes to improve the reproducibility of the work that we publish. This form provides structure for consistency and transparency in reporting. For further information on Nature Research policies, see our Editorial Policies and the Editorial Policy Checklist.

Statistics

For all statistical analyses, confirm that the following items are present in the figure legend, table legend, main text, or Methods section.

n/a	Confirmed	
☐	☒	The exact sample size (*n*) for each experimental group/condition, given as a discrete number and unit of measurement
☐	☒	A statement on whether measurements were taken from distinct samples or whether the same sample was measured repeatedly
☐	☒	The statistical test(s) used AND whether they are one- or two-sided<br>*Only common tests should be described solely by name; describe more complex techniques in the Methods section.*
☒	☐	A description of all covariates tested
☒	☐	A description of any assumptions or corrections, such as tests of normality and adjustment for multiple comparisons
☒	☐	A full description of the statistical parameters including central tendency (e.g. means) or other basic estimates (e.g. regression coefficient) AND variation (e.g. standard deviation) or associated estimates of uncertainty (e.g. confidence intervals)
☒	☐	For null hypothesis testing, the test statistic (e.g. *F*, *t*, *r*) with confidence intervals, effect sizes, degrees of freedom and *P* value noted<br>*Give P values as exact values whenever suitable.*
☒	☐	For Bayesian analysis, information on the choice of priors and Markov chain Monte Carlo settings
☒	☐	For hierarchical and complex designs, identification of the appropriate level for tests and full reporting of outcomes
☒	☐	Estimates of effect sizes (e.g. Cohen's *d*, Pearson's *r*), indicating how they were calculated

Our web collection on statistics for biologists contains articles on many of the points above.

Software and code

Policy information about availability of computer code

Data collection	Phosphorylation-site prediction software NetPhos 2.0 (http://www.cbs.dtu.dk/services/NetPhos).
Data analysis	BD FACSDiva software (BD Biosciences, version 8.0.1) was used to analyze the flow cytometry data.<br>CXP Software (Beckman Coulter, version 2.2) was used to analyze the flow cytometry data.<br>PRISM (GraphPad Software, version 7.0) was for statistical analysis and data presentation.<br>Glomax (Promega, version 1.9.2) was used for luciferase assay data.<br>Zen 2.1 (Zeiss MicroImaging GmbH) are used to view and analize immunofluorescence data.

For manuscripts utilizing custom algorithms or software that are central to the research but not yet described in published literature, software must be made available to editors and reviewers. We strongly encourage code deposition in a community repository (e.g. GitHub). See the Nature Research guidelines for submitting code & software for further information.

Data

Policy information about availability of data

All manuscripts must include a data availability statement. This statement should provide the following information, where applicable:

- Accession codes, unique identifiers, or web links for publicly available datasets
- A list of figures that have associated raw data
- A description of any restrictions on data availability

The data that support the findings of this study are available from the corresponding author upon reasonable request. Source data for Fig. 7e is available in Supplementary Fig 8.

Field-specific reporting

Please select the one below that is the best fit for your research. If you are not sure, read the appropriate sections before making your selection.

☒ Life sciences ☐ Behavioural & social sciences ☐ Ecological, evolutionary & environmental sciences

For a reference copy of the document with all sections, see nature.com/documents/nr-reporting-summary-flat.pdf

Life sciences study design

All studies must disclose on these points even when the disclosure is negative.

Sample size	The sample size for each experiment was five or ten, and described in the figure legend.
Data exclusions	No data has been excluded.
Replication	All experiments were performed with three or more biological replicates.
Randomization	In xenograft and metastasis assay, mice inoculated tumor cells were randomly divided into four groups and injected with the TS120 peptides.
Blinding	In this study, the established cell lines or laboratory animals were analyzed. Blinding was not relevant to our study.

Reporting for specific materials, systems and methods

We require information from authors about some types of materials, experimental systems and methods used in many studies. Here, indicate whether each material, system or method listed is relevant to your study. If you are not sure if a list item applies to your research, read the appropriate section before selecting a response.

Materials & experimental systems

n/a	Involved in the study	
☐	☒	Antibodies
☐	☒	Eukaryotic cell lines
☒	☐	Palaeontology and archaeology
☐	☒	Animals and other organisms
☒	☐	Human research participants
☒	☐	Clinical data
☒	☐	Dual use research of concern

Methods

n/a	Involved in the study	
☒	☐	ChIP-seq
☐	☒	Flow cytometry
☒	☐	MRI-based neuroimaging

Antibodies

Antibodies used

TSPYL5 (N-15)_affinity purified rabbit pAb_sc-98186_Santa Cruz Biotechnology.
PTEN (A2B1)_mouse mAb_sc-7974_Santa Cruz Biotechnology
β-catenin (E-5)_mouse mAb_sc-7963_Santa Cruz Biotechnology
SLUG (A-7)_mouse mAb_sc-166476_Santa Cruz Biotechnology
twist (H-81)_rabbit pAb_sc-15393_Santa Cruz Biotechnology
ZEB1 (H-102)_rabbit pAb_sc-25388_Santa Cruz Biotechnology
CD44 (8E2)_mouse mAb_#5640_Cell Signaling Technology
Sox2 (D6D9) XP®_rabbit mAb_#3579_Cell Signaling Technology
Nanog (1E6C4)_mouse mAb_#4893_Cell Signaling Technology
E-Cadherin (24E10)_rabbit mAb_#3195_Cell Signaling Technology
SNAIL_rabbit pAb_ab63371_Abcam
Anti-ALDH1A1 antibody_EP1933Y_rabbit mAb_ab52492_Abcam
Anti-ALDH1A3 antibody_rabbit pAb_ab80176_Abcam
Anti-Notch2 antibody_rabbit pAb_ab8927_Abcam
CD133_rabbit pAb_18124_Biorbyt
Anti-OCT-4 [POU5F1] Antibody_clone 9E3.2_MAB4305_Millipore.
Anti-N-Cadherin_clone 32/N-Cadherin_610921_BD Bioscience
Vimentin Antibody_SP20_rabbit mAb_MA5-16409_Thermo Fisher Scientific
CD44-APC_IM7_rat mAb_#17-0441-82_eBioscience™
AKT_rabbit pAb_#9272_Cell Signaling Technology
Phospho-AKT (Ser473)_rabbit pAb_#9271_Cell Signaling Technology
Anti-Phospho - (Ser/Thr) antibody_rabbit pAb_ab117253_Abcam
Anti-α-Tubulin antibody_clone B-5-1-2_ascites fluid_T5168_Sigma-Aldrich
HDAC1 (10E2)_mouse mAb_#5356_Cell Signaling Technology
β-actin(C-2)_mouse mAb_sc-8432_Santa Cruz Biotechnology

	GAPDH Antibody (0411)_mouse mAb_sc-47724_Santa Cruz Biotechnology
Validation	TSPYL5 (N-15)_human_WB,IP, IF, ELISA_https://datasheets.scbt.com/sc-98186.pdf<br>PTEN (A2B1)_mouse/rat/human_WB/IP/IF/IHC(P)/ELISA_https://www.scbt.com/ko/p/pten-antibody-a2b1<br>β-catenin (E-5)_mouse/rat/human_WB/IP/IF/IHC(P)/ELISA_https://www.scbt.com/ko/p/beta-catenin-antibody-e-5<br>SLUG (A-7)_mouse/rat/human_WB/IP/IF/IHC(P)/ELISA_https://www.scbt.com/ko/p/slug-antibody-a-7<br>twist (H-81)_mouse/rat/human_WB/IP/IF/ELISA_https://datasheets.scbt.com/sc-15393.pdf<br>ZEB1 (H-102)_mouse/rat/human_WB/IP/IF/ELISA_https://datasheets.scbt.com/sc-25388.pdf<br>CD44 (8E2)_rat/human_WB/IP/IF/FlowCyt_https://www.cellsignal.com/products/primary-antibodies/cd44-8e2-mouse-mab/5640<br>Sox2 (D6D9) XP®_human_WB/IF/FlowCyt_https://www.cellsignal.com/products/primary-antibodies/sox2-d6d9-xp-rabbit-mab/3579<br>Nanog (1E6C4)_human_WB/IHC/IF/FlowCyt_https://www.cellsignal.com/products/primary-antibodies/nanog-1e6c4-mouse-mab/4893<br>E-Cadherin (24E10)_mouse/human_WB/IHC/IF/FlowCyt_https://www.cellsignal.com/products/primary-antibodies/e-cadherin-24e10-rabbit-mab/3195<br>SNAIL_mouse/human_WB/IHC/ELISA_https://www.citeab.com/antibodies/774572-ab63371-anti-snail-slug-antibody<br>Anti-ALDH1A1 antibody_mouse/human_ICC/IF/WB/IP/FlowCyt/IHC-P_https://www.abcam.com/aldh1a1-antibody-ep1933y-c-terminal-ab52492.html<br>Anti-ALDH1A3 antibody_mouse/human_WB/ELISA_https://www.citeab.com/antibodies/709869-ab80176-anti-aldh1a3-antibody-n-terminal<br>Anti-Notch2 antibody_human_IHC-P/WB_https://www.abcam.com/notch2-antibody-ab8927.html<br>CD133_rat/human_IHC-P/WB_https://www.citeab.com/antibodies/1077654-orb18124-cd133-antibody<br>Anti-OCT-4 [POU5F1] Antibody_rat/human_WB/ELISA_https://www.citeab.com/antibodies/226452-mab4305-anti-oct-4-antibody-clone-9e3-2<br>Anti-N-Cadherin_human/mouse/rat_WB/IP/IF_https://www.labome.com/product/BD-Biosciences/610921.html<br>Vimentin_Antibody_human_WB/IHC/ICC/IF/FlowCyt_https://www.thermofisher.com/antibody/product/MA5-16409.html?ef_id<br>CD44-APC_mouse/human_FlowCyt_https://www.thermofisher.com/antibody/product/CD44-Antibody-clone-IM7-Monoclonal/17-0441-82<br>AKT_human/mouse/rat_WB/IP/IF/FlowCyt_https://www.cellsignal.com/products/primary-antibodies/akt-antibody/9272<br>Phospho-AKT(Ser473)_human/mouse/rat_WB/IP/IF/FlowCyt_https://www.cellsignal.com/products/primary-antibodies/phospho-akt-ser473-antibody/9271<br>Anti-Phospho - (Ser/Thr) antibody_Species independent_ELISA/Dotblot_https://www.abcam.com/phospho-serthr-antibody-ab117253.html<br>Anti-α-Tubulin antibody_human/mouse/rat_IF/RIA/WB_https://www.sigmaaldrich.com/catalog/search?term=t5168<br>HDAC1 (10E2)_human/mouse/rat_WB/IP_https://www.cellsignal.com/products/primary-antibodies/hdac1-10e2-mouse-mab/5356<br>β-actin_human/mouse/rat_WB/IP/IF/IHC(P)/FCM/ELISA_https://www.biocompare.com/9776-Antibodies/249932-Actin-C2/<br>GAPDH Antibody (0411)_human_WB/IP/IF/IHC(P)_https://www.scbt.com/p/gapdh-antibody

Eukaryotic cell lines

Policy information about cell lines

Cell line source(s)	A549 (ATCC® CCL-185), NCI-H460 [H460] (ATCC® HTB-177), NCI-H358 [H358] (ATCC® CRL-5807), NCI-H2009 [H2009] (ATCC® CRL-5911), HepG2 [HEPG2] (ATCC® HB-8065), Huh7(JCRB0403), KP3(JCRB0178.0), PANC-1 (ATCC® CRL-1469)
Authentication	Cell lines were obtained from ATCC or JCRB Cell Bank.<br>https://www.atcc.org/; https://cellbank.nibiohn.go.jp/~cellbank/en/search_res_det.cgi?ID=2013.
Mycoplasma contamination	Negative
Commonly misidentified lines<br>(See ICLAC register)	None

Animals and other organisms

Policy information about studies involving animals; ARRIVE guidelines recommended for reporting animal research

Laboratory animals	BALB/c female nude mice, female, 5 weeks of age
Wild animals	N/A
Field-collected samples	N/A
Ethics oversight	The methods were performed in accordance with relevant guidelines and regulations and approved by the Animal Care and Use Committee (IACUC) of the Korea Research Institute of Bioscience & Biotechnology (Approval No: KRIBB-AEC-19162).

Note that full information on the approval of the study protocol must also be provided in the manuscript.

Flow Cytometry

Plots

Confirm that:

☒ The axis labels state the marker and fluorochrome used (e.g. CD4-FITC).

☒ The axis scales are clearly visible. Include numbers along axes only for bottom left plot of group (a 'group' is an analysis of identical markers).

☒ All plots are contour plots with outliers or pseudocolor plots.

☒ A numerical value for number of cells or percentage (with statistics) is provided.

Methodology

Sample preparation	For fluorescence-activated cell sorting (FACS), 2 × 10^6 cells were collected and then stained with ALDH substrate or APC-conjugated mouse anti-human CD44 mAb according to the manufacturer's instructions. The cells were washed twice with PBS containing 1% FBS and suspended in 1 ml wash buffer. These cells were sorted by BD FACSAria cell sorter (BD Biosciences). For the analysis of ALDH1 activity or CD44 expression, 1 × 10^5 cells were stained with ALDH substrate or APC-CD44 mAb and analysed by Cytomics FC 500 (Beckman Coulter).
Instrument	FACSAria cell sorter (BD Biosciences)<br>Cytomics FC 500 (Beckman Coulter)
Software	BD FACSDiva software<br>CXP analysis 2.2 software
Cell population abundance	Sorted cells (1 x 10^5) were obtained with a purity of 99% by setting the negative and positive gates to the level of 5% using sorting system.
Gating strategy	DEAB (ALDH inhibitor)-treated cells or isotype control IgG-stained cells were used as negative controls for gating.

☒ Tick this box to confirm that a figure exemplifying the gating strategy is provided in the Supplementary Information.
